# Draft Genome Sequences of Three *Escherichia coli* Strains with Different *In Vivo* Pathogenicities in an Avian (Ascending) Infection Model of the Oviduct

**DOI:** 10.1128/genomeA.00399-15

**Published:** 2015-05-07

**Authors:** Rikke Heidemann Olsen, Ida Cecilie Naundrup Thøfner, Susanne Elisabeth Pors, Henrik Christensen, Magne Bisgaard, Jens Peter Christensen

**Affiliations:** a University of Copenhagen, Department of Veterinary Disease Biology, Frederiksberg, Denmark; b Viby Sjælland, Denmark

## Abstract

Here, we present three draft genome sequences of *Escherichia coli* strains that experimentally were proven to possess low (strain D2-2), intermediate (Chronic_salp), or high virulence (Cp6salp3) in an avian (ascending) infection model of the oviduct.

## GENOME ANNOUNCEMENT

*Escherichia coli* is associated with a variety of extraintestinal infections in poultry, collectively termed colibacillosis. *E. coli* strains causing disease in poultry are designated avian-pathogenic *E. coli* (APEC) strains; however, not all APEC strains are equally virulent. Most strains of *E. coli* are opportunistic pathogens, whereas a limited number of APEC strains might act a primary pathogen (1). Here, we present three draft genome sequences of *E. coli* strains that experimentally were proven to process low (strain D2-2), intermediate (Chronic_salp), and high virulence potential (Cp6salp3) in an avian experimental infection model (2).

*E. coli* strain D2-2 represents an intestinal commensal strain from a healthy chicken, strain Cp6salp3 originates from the salpinx of a chickens with salpingitis (field monoclonal outbreak), and Chronic_salp was obtained from the salpinx of a clinical case of avian salpingitis (not outbreak related).

All three genomes were sequenced by the Illumina paired-end method (MiSeq 150, 30× coverage) using a paired-end library with an average read length of 2 × 150 bp. CLC Genomics Workbench version 7.0.4 was used for *de novo* assembly and trimming of the genomes. The total size of assembly/mean contig size of the genomes for D2-2, Chronic_salp, and Cp6salp3 were 5,681,572/34,742, 5,028,456/9,922, and 5,095,283/21,448, resulting in 560, 135, and 227 contigs and an average G+C content of 50.4%, 50.4%, and 50.5%, respectively.

The NCBI prokaryotic pipeline genome automatic annotation pipeline (PGAAP) was employed for annotation.

The three genomes for D2-2, Chronic_salp, and Cp6salp3) consist of 5,982, 5,069, and 5,234 putative genes, respectively, of which 5,590, 4,861, and 5,017 are protein encoding (coding sequences [CDSs]).

PathogenFinder 1.1 (3) was used to estimate the number of pathogenic families for each genome. Cp6salp3 contains significantly (959) more pathogenic families than D2-2 and Chronic_salp (623 and 523, respectively). In addition, all three strains had a prediction of >86% for being human pathogens.

A total of 58 sequences of genes associated with extraintestinal virulence were extracted from the Virulence Factors of Pathogenic Bacteria (VFPB) database (4) and blasted against the three genomes by applying the BLAST Ring Image Generator (BRIG) (5). Both Chronic_salp and D2-2 lacked *iutA*, and D2-2 also lacked *iroN* (genes suggested as minimal predicators for APEC [6]), in addition to *ups* and *tsh*. Cp6Salp3 was the only genome harboring *papA, tia, iucB*, and *iucC*. Besides these genes, Cp6salp3 and Chronic_salp had identical virulence profiles, harboring 37/58 and 32/58 genes included in the analysis, respectively.

In conclusion, these three draft genome sequences are from the first avian *E. coli* isolates with documented experimental *in vivo* pathogenicity. In the future, as more strains will be evaluated for their *in vivo* pathogenicity in the salpingitis model, the three reported genome sequences may be used for genomic comparison to identify genomic traits associated with strains of low, intermediate, or high virulence potential in relation to the oviduct. A more detailed genomic investigation of the three genomes will be presented in a future publication.

### Nucleotide sequence accession numbers. 

The three whole-genome shotgun projects have been deposited at DDBJ/EMBL/GenBank under the accession numbers JYEE00000000 (Chronic_salp), JYEF00000000 (D2-2), and JYED00000000 (Cp6salp3). The versions described in this paper are all first versions.
